# Organic–Inorganic Hybrid Ladder-like Polysilsesquioxanes as Compatibilized Nanofiller for Nanocomposite Materials

**DOI:** 10.3390/molecules29245832

**Published:** 2024-12-11

**Authors:** Dominique Mouysset, Marion Rollet, Emily Bloch, Stéphane Gastaldi, Eric Besson, Trang N. T. Phan

**Affiliations:** 1 Aix Marseille Univ, CNRS, Chemistry Department, Institute of Radical Chemistry (ICR), 13397 Marseille, France; dominique.mouysset@univ-amu.fr (D.M.); marion.rollet@univ-amu.fr (M.R.); stephane.gastaldi@univ-amu.fr (S.G.); 2 Aix Marseille Univ, CNRS, Chemistry Department, Laboratory of Divided Materials, Interfaces, Reactivity, Electrochemistry (MADIREL), 13397 Marseille, France; emily.bloch@univ-amu.fr

**Keywords:** ladder polysilsesquioxane, PEG-grafted LPSQ, nanocomposites

## Abstract

Nanocomposite materials composed of an organic matrix and an inorganic nanofiller have been the subject of intense research in recent years. Indeed, the synergy between these two phases confers improved properties thanks to an increased surface–volume ratio, which reinforces the interactions between the particles and the polymer matrix. These interactions depend on many factors such as the shape, size and dispersion of the nanoobjects. Polysilsesquioxanes (PSQs) are a silicon polymer family that offers different sizes, shapes and structures and possesses ceramics properties (i.e., high thermal and/or oxidative resistance and high chain rigidity), thanks to the siloxane backbone. In this article, we propose to incorporate polymer-grafted ladder polysilsesquioxanes (LPSQs) as nanofillers in thermoplastic matrices. Chloride-functionalized LPSQs were synthesized from two different precursors and thoroughly characterized by ^1^H, ^13^C and ^29^Si NMR, as well as by SEC and WAXS. The well-defined LPSQ was then converted into an azide analog. The resulting hybrid material was functionalized with poly(ethylene glycol) (PEG) chains and incorporated into poly(ethylene oxide) or poly(methyl methacrylate) matrices. We found that the viscoelastic properties of the nanocomposite materials were impacted by plasticizing or the reinforcement effect depending on the grafted PEG chain length.

## 1. Introduction

With a global production of more than 300 million tons, synthetic polymers occupy an increasingly important place in many objects and materials in everyday life. They are found in all sectors as commodity materials (packaging, transport, sports and leisure, etc.) or as specialty polymers where they perform more “noble” functions in fields such as electronics, optics, biomedical and cosmetics. In recent decades, new hybrid materials have been developed. These materials aim to exploit the complementary properties of their components or to create synergy between them; we then speak of composites. In other words, a composite material is a material for which the combination of basic components leads to a material having properties that none of the components taken separately has. They can be defined as a material resulting from the association of a continuous phase, the matrix, and a dispersed phase, strengthening. Depending on the composition of composite materials, they can have many properties and therefore many advantages. Composite materials allow almost infinite freedom of assembly, which makes it possible to create on demand materials with the desired properties depending on the product manufactured. Today, there are a large number of composite materials that are generally classified into three families depending on the nature of the matrix, which can be ceramic, metallic or organic. Organic-based composites are the most important class of these materials thanks to their advantages such as ease of implementation, low cost, resistance to corrosion and their major advantage compared to metallic compounds, their low weight. These materials are prepared by adding fillers, generally inorganic for an organic matrix, in the form of a particle or fiber. However, the efficiency of the performances expected by the addition of these charges is linked to the quantity of surface area of the interfaces, generally requiring a significant concentration of charge. The properties of the polymer matrix in terms of processability are strongly affected. This problem found a solution in the early 1990s, with the pioneering work of Toyota researchers by the incorporation of a small amount (a few percent by mass) of lamellar clay in a polyamide [1]. These new composite materials, named nanocomposites because at least one of the phases has at least one dimension less than 100 nanometers, have seen their mechanical properties greatly improved. Indeed, the very small dimensions of these nanofillers allow increasing the surface-to-volume ratio, reinforcing the degree of particle–matrix interactions.

Thus, the nanocomposites’ properties and the filler/polymer interactions depend on several factors such as the shape and size, the quantity and dispersion quality, the orientation and distribution of the nanoobjects. Of course, all are connected together and to ensure a good transfer of the properties of the nanomaterial to the polymeric matrix, it is necessary to control its shape (for a high aspect ratio) [2] and the interface [3]. For the latter, generally, a modification of the nanofiller by macromolecules, identical or compatible, to those of the matrix is carried out in order to increase their affinities and the quality of dispersion.

Among the different fillers, silsesquioxane (SSQs) or polysilsesquioxane (PSQ) have attracted particular attention because they can be functionalized with a large diversity of organic groups and are inorganic materials with a high compatibility with organic materials. PSQs are of general formula [R-SiO_1,5_] and can be prepared in many shapes, such as random, ladder (LPSQs), lamellar, cage (POSS) or partial cage structures [4,5,6].

Considered as the smallest hybrid particles of silica, POSS was certainly the most SSQs used for the preparation of nanocomposite materials [7]. They can be incorporated in the polymer matrix as non-functional (reinforcement agent), monofunctional (polymer end-capping agent) or multifunctional (crosslinking agent) POSS. Similarly, we have recently prepared a new alkoxyamine-functionalized lamellar PSQ hybrid material, exfoliated it and used it both as a filler and polymerization initiator in a grafting from strategy. The presence of polysilsesquioxane in the composites had an impact on their viscoelastic properties, demonstrating the reinforcement of the polymeric matrix by the lamellae [8]. LPSQ is another SSQ with a tailorable and controlled structure described in these last few years because it addresses some POSS limitations (solubility, low molecular weight...) [9]. In addition to the thermal/chemical stability of the siloxane backbone, the double-stranded structure contributes to the robustness of the LPSQ backbone. The work of Lee is a good example [10]. They systematically studied the effect of the SSQ structure on the mechanical properties of SSQ-reinforced polymer film. The results suggest that the highly ordered and one-dimensional LPSQ, with a higher aspect ratio, leads to a better enhancement in the mechanical properties than random and POSS SQs. In these composites, the SSQ structures were covalently linked to the polymer matrix via the photopolymerization of methacrylate groups grafted on SSQ structures and an acrylate resin used as the polymer matrix. Another approach to improve the compatibility of LPSQ and polyimide was studied by Kim et al., which is based on the hydrogen-bonding interactions between PEG-grafted LPSQ and the carboxylic acid groups of polyimide [11]. Nevertheless, the LPSQ used was also functionalized with methacrylate groups and copolymerized by UV-curing with PEG-dimethacrylate presence in the polyimide composite formulation. As mentioned above, one main strategy to improve the dispersion of fillers such as silica nanoparticles within polymeric matrices is to functionalize their surfaces with polymers identical or compatible to those of the matrix [12]. This approach is easy to implement and an efficient way to enhance the compatibility between the nanofiller and organic polymer matrix, but it has rarely been exploited with LPSQs in the literature.

In the aim to enlarge the development of LPSQ as a nanofiller and simplify its composite elaboration while ensuring their good dispersion within the commercial polymer matrix, we studied, in the present work, the synthesis of well-defined poly(ethylene glycol) (PEG)-grafted LPSQs and their use as filler in two different commercial polymer matrices, namely poly(ethylene oxide) (PEO) and poly(methyl methacrylate) (PMMA). PEO is one of the most studied polymer electrolytes for lithium battery application [13]. This is because PEO presents several advantages such as good flexibility, ease of processing, excellent interfacial stability with lithium electrode and the formation of complexes with lithium salts. However, the commercialization of PEO-based electrolytes in lithium battery is difficult because their room-temperature (RT) ionic conductivity is too low due to the high degree of crystallization of PEO at RT that reduces the chain mobility and, hence, lithium mobility as well as ionic conductivity. We expect that the incorporation of PEG-grafted LPSQ allows for reducing the PEO crystallinity while maintaining or improving its mechanical properties. Therefore, it could be an interesting approach to increase the ionic conductivity of PEO-based electrolytes. On the other hand, blending polymers is an efficient and economic method to develop new polymeric materials. PEO/PMMA is a partially miscible crystalline/amorphous blend, which has attracted much interest [14,15,16,17,18,19]. Currently, there are no papers investigating the blends of PMMA and grafted PEO and their viscoelastic properties. So, it could be interesting to study the behavior of PEG-grafted LPSQ and PMMA blends. For these reasons, we first synthesized chloride-functionalized LPSQ from two different precursors. The well-defined LPSQ was chosen for conversion into an azide analog before grafting with PEG chains by the “grafting to” approach. Secondly, the resulting PEG-grafted LPSQ hybrid materials were blended at different contents with PEO or PMMA. It is expected that the presence of grafted PEG on LPSQ will increase their affinities/compatibilities with the polymer matrix, and hence the quality of dispersion, in order to ensure a good transfer of the properties of the hybrid material to the polymeric matrix.

## 2. Results and Discussion

### 2.1. Synthesis of Ladder Hybrid Materials

**Scheme 1:**
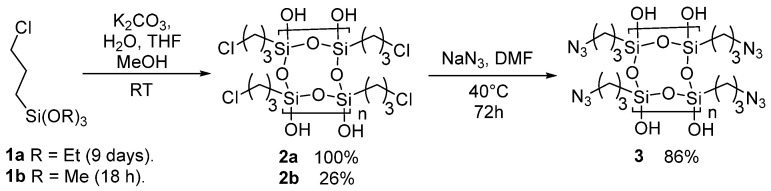
Synthesis of functionalized ladder materials.

The synthesis of functionalized ladder-like polysilsesquioxanes (LPSQs) was implemented from 3-chloropropyl trialkoxysilane **1a** and **1b** in order to assess the impact of their alkoxy groups, i.e., ethoxy or methoxy, on the features of the LPSQs (Scheme 1) [20]. **1a** or **1b** was stirred at room temperature in a water/THF mixture in the presence of a catalytic amount of K_2_CO_3_ (Scheme 1) [21]. In the case of **1a**, MeOH (0.5 equiv.) was added for triggering silanol formation. After only 18 h, NMR monitoring showed the completion of the polymerization of **1b**. This result is in agreement with a fast hydrolysis of trimethoxysilane moiety. The presence of insoluble PSQ in the crude solution resulted from random polymerization; its filtration yielded LPSQ **2b** in 26% yield. As expected, in the case of **1a**, the reaction was much slower, and 9 days were needed to reach 96% of the polymerization of **1a** (NMR monitoring). Under these slow polymerization conditions, no insoluble material, i.e., random polymerization, was detected. After work-up, **2a** was obtained quantitatively.

The solubility of **2a** and **2b** in several organic solvents allows performing the liquid NMR analysis in CDCl_3_. The ^1^H and ^13^C spectra confirmed the integrity of the organic moieties under the sol–gel process conditions, as shown by the presence of characteristic signals at 3.52 (CH_2_Cl) and 0.79 ppm (CH_2_Si) recorded for the two LPSQs.

Solid-state ^29^Si NMR (SS NMR) is one of the key analyses for asserting the ladder structure of the PSQ. Indeed, highlighting the difference between the ratio of T^1^ (R*Si*(OH)_2_OSi), T^2^ (R*Si*(OH)(OSi)_2_) and T^3^ (R*Si*(OSi)_3_) siloxane structures allows revealing the level of siloxane bond formation [9]. The analysis of the ^29^Si NMR spectra for **2a** and **2b** showed the presence of T^1^, T^2^ and T^3^ signals of very weak, weak and strong intensity, respectively (Figures S1 and S2, Supplementary Materials). PSQ **2a** and **2b** exhibited a high degree of condensation (DOC) of 98.8 and 97.5%, consistent with an LPSQ structure [9].

The second key analysis is the Fourier-transform infrared (FTIR) spectroscopy. Indeed, a characteristic double peak at 1100 and 1026 cm^−1^ is observed and attributed to the asymmetric and symmetric stretching vibration of Si–O–Si, which is directly linked to the LPSQ structure (Figure S3, Supplementary Materials) [22]. Furthermore, the absence of peaks corresponding to the silanol group (Si-OH) at 3500 cm^−1^ and 960 cm^−1^ was in accord with ^29^Si SS NMR, which exhibited a very weak T^1^ signal and weak peak for T^2^ in the case of **2a**, confirming the high level of condensation of the silanol to form siloxane bridges.

**Figure 1:**
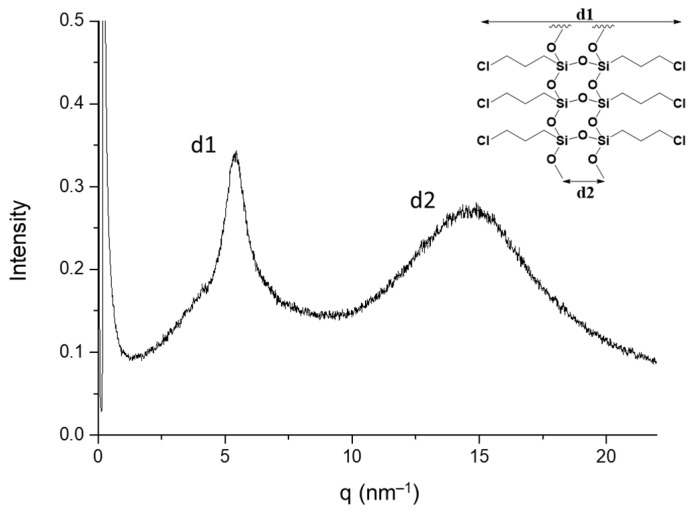
WAXS measurement of LPSQ **2a**.

Powder wide-angle X-ray scattering (WAXS) analysis was carried out on **2a** and gives a good insight into the structure of the polysilsesquioxane. The WAXS pattern (Figure 1) exhibits two maxima at 5.4 and 14.8 nm^−1^. The first maximum corresponds to distance d1 = 1.16 nm, attributed to the chain-to-chain distance, and the second d2 = 0.42 nm is related to the average thickness of the double-stranded siloxane backbone. Whereas random polysilsequioxanes generally showed a unique broad peak due to low regularity and POSS displayed sharp peaks, these results are in accordance with ladder-like polysilsesquioxane [23].

SEC analysis was used to determine the M_w_. The LPSQ **2b**, prepared from trimethoxysilane **1b**, had a slightly lower M_w_ of 8200 g mol^−1^ than that measured for **2a** of 10,500 g mol^−1^ and a comparable dispersity (Ð) of 2.4 and 2.5, respectively.

These analyses clearly pointed to a well-defined structure for **2a**. The slower polycondensation with the triethoxysilane **1a** provided an LPSQ with a low level of defects, a higher molecular weight and a higher yield. Therefore, LPSQ **2a** was used for the remainder of this study.

The second step in the preparation of the hybrid material was the introduction of an azide function via a nucleophilic substitution, so that in the next step, a poly(ethylene glycol) chain could be introduced via a click reaction. A solution of **2a** in DMF was stirred at 40 °C for 72 h in the presence of 1.5 equivalents of NaN_3_. N_3_-LPSQ **3** was obtained with a yield of 86%. ^1^H and ^13^C NMR confirmed a total conversion as no CH_2_Cl signals were detected and new signals corresponding to CH_2_N_3_ appeared at 3.28 and 53.5 ppm, respectively. The FTIR analysis also established the presence of an azide function (2090 cm^−1^) and the stability of the ladder structure under substitution conditions, thanks to the two peaks at 1096 and 1040 cm^−1^. It is noteworthy that the ^29^Si NMR spectrum now displayed a complete disappearance of the T^1^ signal, as shown by the DOC of 99.7%, calculated from the integration of T^n^ signals. It is likely that the aforementioned substitution conditions also favored the formation of the missing siloxane bonds in the ladder structure. At this point, silanol polycondensation can be considered complete.

**Scheme 2:**
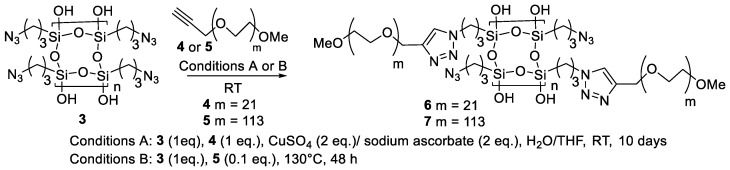
Synthesis of poly(ethylene glycol)-grafted ladder materials.

The last step in the synthesis of the ladder-based hybrid material was the grafting of a polyethylene glycol (PEG) chain through a click reaction between a triple bond and an azide function. This reaction was performed with two propargylated PEG with molecular weights of 1000 g mol^−1^ (**4**) and 5000 g mol^−1^ (**5**) (Scheme 2). Two different experimental conditions were implemented to form the 1,2,3-triazole ring. In the first approach, the copper sulfate/sodium ascorbate couple was used to catalyze the click reaction [24,25], while the second method involved a thermal activation to enable the [2+3] cycloaddition [24].

In both cases, the reaction advancement was monitored by ^1^H NMR with the disappearance of the Csp-H signal at 2.4 ppm from the triple bond of **4** or **5**. It is worth pointing out that the ^13^C NMR analysis of **6** (PEG1-LPSQ) and **7** (PEG5-LPSQ) showed that the copper-catalyzed cyclization led only to 1,4-disubstituted 1,2,3-triazole (C quaternary = 143.5 ppm, Csp^2^-H = 123.1 ppm) whereas the thermal conditions provided a 1:1 mixture of 1,4- and 1,5-disubstituted rings (C quaternary and Csp^2^H = 134.5 ppm). TGA analysis was used to determine the grafting yield of the PEG chains, which was 56 mol % for **6** and 4 mol % for **7** as well as the weight fraction of PEG in these hybrid materials, which was 80 wt% and 61 wt%, respectively. The calculations are detailed in Figures S4 and S5 (Supplementary Materials). Although the grafting did not involve all the azide functions, it can be noted that, in the case of PEG1-LPSQ (**6**), the characteristic FTIR absorption band attributed to the azide group at 2090 cm^−1^ was weak compared to that which should be expected while that of PEG5-LPSQ (**7**) is still observable (Figure S6a,b, Supplementary Materials). Concerning the ^13^C NMR analysis, the same observation was made for PEG1-LPSQ (**6**) and PEG5-LPSQ (**7**); in other words, the signal corresponding to CH_2_-N_3_ around 53 ppm is weak or even undetectable for the PEG1-LPSQ (**6**) compound and well present in the spectra of PEG5-LPSQ (**7**) (Figure S7a,b, Supplementary Materials). Differential scanning calorimetry (DSC) was used to analyze the thermal behavior of grafted PEG and look at how the presence of the LPSQ, covalently linked to PEG chains, affects the melting temperature (T_m_) and the crystallinity degree ($X_{c}$) of PEG. The pristine PEG of 1000 g mol^−1^ and 5000 g mol^−1^ exhibit a T_m_ of 38.7 and 64.3 °C, respectively. The PEG-grafted LPSQ showed a T_m_ of 36.5 and 57.3 °C for **6** and **7**, respectively, which are slightly lower than those of the corresponding pristine PEG. From the heat fusion of the DSC thermogram (Figures S8 and S9, Supplementary Materials), the crystallinity degree of PEG was calculated using Equation (1) and reported in Table 1. We observe a significant reduction in $X_{c}$ of the grafted PEG compared to the pristine PEG of the same molar mass and this trend is more pronounced with the PEG of low molecular weight. Since the mobility of the PEG chains is restricted by virtue of being covalently bonded to the inorganic LPSQ, this inhibits the crystallization of the PEG chains and thus reduces the crystallinity degree of the PEG. In consequence, the shorter the PEG chains, the higher the fraction of the restricted segments and the lower the crystallinity degree (Table 1).

### 2.2. Elaboration and Characterization of Composite Materials Based on PEG-Grafted LPSQ

The PEG-grafted LPSQ hybrid materials were used as nanofiller and blended with poly(ethylene oxide) (PEO) or poly(methyl methacrylate) (PMMA) as matrices to elaborate different composites using the solvent casting process. Polymer nanocomposites can be defined as a class of materials where one or more phases with nanoscale dimensions are embedded in a polymer matrix [26]. Since the size of LPSQs are smaller than 4 nm (according to their molar mass), the resulting blends between PEG-grafted LPSQ and PEO or PMMA matrices can be qualified as polymer nanocomposites. Due to the small size of LPSQs, their low contrast with polymer matrix and their dilution (≤2.5 wt%) in the blends, it is difficult to analyze their dispersion and structure in the blends by WAXS as when carried out for LPSQs alone. Indeed, WAXS of blends containing PEG-grafted LPSQ and polyimide with a weight fraction of PEG-grafted LPSQ lower than 50 wt% did not show proof of the dispersion of functional LPSQ in the polyimide matrix [10].

#### 2.2.1. DSC Analysis

**Figure 2:**
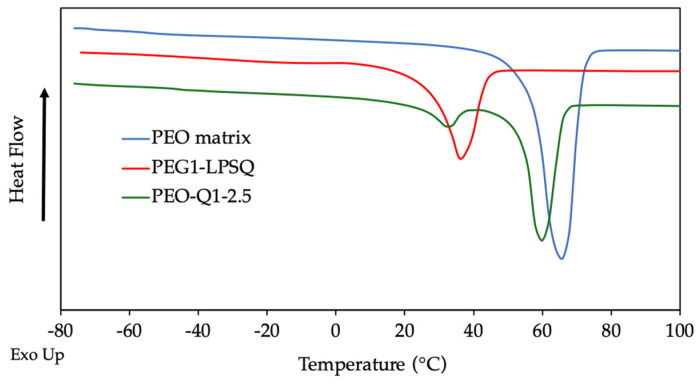
DSC thermograms during the second heating run for PEO matrix (blue solid line), PEG1-LPSQ (red solid line) and PEO-Q1-2.5 composite containing 2.5 wt% of silica (green solid line).

**Figure 3:**
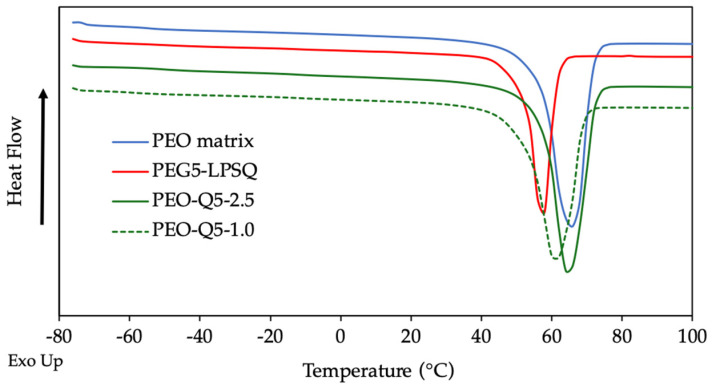
DSC thermograms during the second heating run for PEO matrix (blue solid line), PEG5-LPSQ (red solid line), PEO-Q5-2.5 composite containing 2.5 wt% of silica (green solid line) and PEO-Q5-1.0 composite containing 1 wt% of silica (green dash line).

The thermal behavior of the nanocomposite materials was investigated using DSC. The DSC traces were taken during the 2nd heating to remove any thermal history. Figure 2 and Figure 3 show the heating thermograms for PEO matrix, PEG-grafted LPSQ and their composites. Table 1 summarizes glass transition temperature (T_g_), T_m_ and X_c_ values obtained from the thermograms. As shown in Figure 2 and Figure 3, a single T_g_ was observed for the resulting composites containing PEG1-LPSQ and PEG5-LPSQ, suggesting that PEO and PEG-grafted LPSQ are fully miscible in an amorphous phase. The high miscibility of the system is ascribed to the PEG-based LPSQ grafted chains having the same repeat unit structure as the PEO matrix. In the composites prepared with the same nanofiller e.g. PEG5-LPSQ, we observed a reduction in T_g_ with an increase in PEG5-LPSQ content (Table 1). This trend was also reported for nanocomposite electrolytes composing of PEO mixed with POSS functionalized with PEG of 637 g mol^−1^ [27]. This suggests that the grafted PEG chains in the LPSQ act as internal plasticizers to PEO. On the other hand, the composites containing the same weight fraction of silica (e.g., 2.5 wt%) but prepared with different PEG-grafted LPSQ didn’t exhibit the same T_g_. The PEO-Q1-2.5 composite shows a higher T_g_ (–45.9 °C) value than that of PEO-Q5-2.5 (–51.2 °C) composite (Table 1) suggesting that plasticizing character of short PEG chains (1000 g mol^−1^) is less than that of long PEG chains (5000 g mol^−1^).

Concerning the melting of PEO in the nanocomposites, as seen in Figure 2 and Figure 3, both the T_m_ and melting enthalpy slightly decrease when PEG-LPSQ nanofillers are incorporated into PEO. Furthermore, we noted an additional small peak appears on the heating thermogram of the PEO-Q1-2.5 composite (Figure 2), indicating the formation of a new crystalline phase. This peak is very close to the peak of PEG5-LPSQ. This finding was already reported in the literature for the mixture of PEG-grafted POSS with a PEO matrix [27]. According to this paper, the concentration of LPSQ moieties in the new crystalline phase (LPSQ-rich phase) would be greater than that of the crystalline phase at a higher temperature (PEO-rich phase).

The PEO/PMMA blends were largely studied in the literature because of their hydrophilic/hydrophobic character and their compatibility at certain compositions [16,17,18,19]. According to the work of Dionisio et al., PEO is miscible with PMMA up to a content of 25% of PEO in the blend [28]. The behavior of polymer blends, in particular the degree of miscibility, is strongly dependent on the molecular weight, the tacticity of PMMA and on the thermal history previous to the measurements. Decreases in both T_m_ of PEO and T_g_ of PMMA were generally observed for PEO/PMMA blends [29,30,31]. The DSC thermograms of PMMA-based composites and the PMMA matrix are shown in Figures S10–S12, and DSC data are reported in Table 1. The PMMA-Q1-2.5 composite shows an endothermic melting peak at T_m_ = 28 °C corresponding to the crystalline phase of PEG (Figure S11) and two glass transition temperatures at –43 °C (PEG phase) and 97 °C (PMMA phase). Since the percentage of PEG in this composite is around 32 wt%, which is higher than that of the low limit of PEO content for a miscibility blend, the thermal behavior of the blend is mainly determined by the PEG component, and both T_m_ and X_c_ are less affected by the PMMA phase (Table 1, Entry 9). However, the T_g_ of the PMMA phase is lower than that of pristine PMMA (T_g_ = 117 °C) (Figure S10, Supplementary Materials), indicating the plasticization of PMMA by short PEG chains grafted on LPSQ. The PMMA-Q5-2.5 composite containing 10 wt% of grafted PEG shows a tiny melting peak at 47 °C and a unique T_g_ at −13 °C (Figure S12). According to the literature [15,16,18,28], at this PEO content, the blend is completely miscible showing a single T_g_. The T_g_ observed for our sample is very far away from the expected T_g_ value (T_g_ = 89 °C) for a blend comprising 10 wt% of PEO and 90 wt% of PMMA [28]. Nevertheless, in our case, the PEO chains are grafted to the LPSQ structure, and this could greatly modify the behavior of polymer blends.

#### 2.2.2. Rheology Analysis

**Figure 4:**
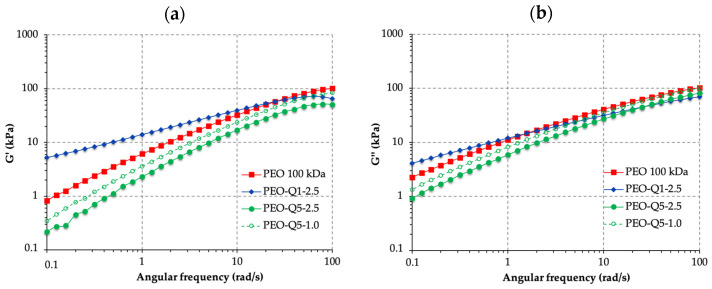
(**a**) Storage modulus (G’) and (**b**) loss modulus (G”) versus angular frequency of PEG-grafted LPSQ/PEO composites compared to neat PEO matrix of 100 kg mol^−1^. Measurements were performed at 80 °C with a strain amplitude of 1%.

**Figure 5:**
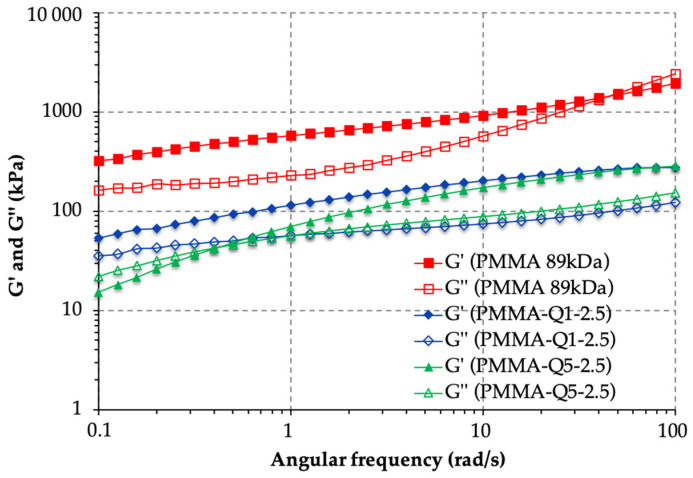
Storage (G’) and loss (G”) modulus versus angular frequency of PEG-grafted LPSQ/PMMA composites compared to neat PMMA matrix of 89 kg mol^−1^. Measurements were performed at 150 °C with a strain amplitude of 1%.

Storage modulus (G’) and loss modulus (G”) are measured for all nanocomposites prepared with two different PEG-grafted LPSQs and their matrices (PEO and PMMA) by dynamic oscillatory measurements (Figure 4a,b and Figure 5). The storage modulus of PEO-based nanocomposites presents a monotonic increase for all frequencies, including pure PEO. For pure PEO and its nanocomposites, the increment of frequency promotes the modulus because the polymer chains do not have enough time to relax at high frequencies. On the other hand, a low frequency or long time allows the relaxation of polymer chains to lower the modulus.

At 80 °C, pure PEO and PEO nanocomposites are under the melting state. As a consequence, the pure PEO melt exhibits a liquid-like behavior (G” > G’) in all frequencies (Figure 4), while PEO composites present two different behaviors dependent on the type of functional LPSQ used. The PEO-Q1-2.5 nanocomposite prepared with the LPSQ functionalized with short PEG chains (1000 g mol^−1^) shows solid-like behavior (G’ > G”) from 0.1 to 80 rad/s, while PEO-Q5-2.5 composite prepared with the LPSQ functionalized with longer PEG chains (5000 g mol^−1^) shows a liquid-like behavior for all frequencies. In addition, we note that the values of storage modulus vary in the order G’ (PEO-Q1-2.5) > G’ (pure PEO) > G (PEO-Q5-1.0) > G’ (PEO-Q5-2.5) for almost all frequencies. These results suggest that PEG1-LPSQ is more efficient for the reinforcement of PEO matrix. Figure S13 (Supplementary Materials) shows the frequency dependence of tan δ = G”/G’ for nanoassociated (tan δ > 3), weakly associated (1 < tan δ < 3) and strongly associated (tan δ < 1) dispersed functional LPSQ molecules [32]. Here, we think that when the grafted PEG chains are short (M_n_ < M_c_, where M_c_ of 4000 g mol^−1^ is the critical entanglement molecular weight of PEO) [33], the LPSQ molecules act as entangled attractors, resulting in higher entanglements near the surface of the LPSQ molecules, which reinforce the PEO matrix. On the contrary, when the grafted PEG chains are sufficiently long (M_n_ > M_c_), grafted PEG chains act as entangled attractants, which lead to only PEG/PEO entanglements and PEG/PEO interactions, resulting in a plasticizing effect for the PEO matrix.

It is obviously apparent from Figure 5 that the PMMA/PEG-LPSQ nanocomposites show a lower value of G’ and G” compared to the pure PMMA. A decrease of one to one-and-a-haft decades in both the elastic (G’) and loss (G”) modulus lower than that of the pure PMMA is observed for all frequencies. The PMMA-Q1-2.5 composite exhibits a solid-like behavior for all frequencies, while the PMMA-Q5-2.5 composite is liquidlike at low frequencies. The transition from liquidlike to solid-like behavior occurs at the crossover frequency of G’ and G” of 0.4 rad s^−1^. This result illustrates that the dynamics of PMMA are significantly enhanced owing to the plasticizing nature of the PEG chains for the PMMA matrix. This effect increases with the grafted PEG chain length.

## 3. Materials and Methods

### 3.1. Materials

(3-Chloropropyl)trimethoxysilane (>97%), (3-Chloropropyl)triethoxysilane (95%), sodium azide (>99%), propargyl bromide (80% in toluene), sodium hydride (60% dispersion in oil), ethylenediaminetetraacetic acid (EDTA), poly(ethylene glycol) methyl ether of 1000 g mol^−1^ (PEG1) and 5000 g mol^−1^ (PEG5), poly(ethylene oxide) (PEO) of 100,000 g mol^−1^ and poly(methyl methacrylate) (PMMA) of 89,000 g mol^−1^ were purchased from Sigma-Aldrich (Saint Louis, MO, USA) and used as received. All solvents such as tetrahydrofuran (THF), dichloromethane (DCM), dimethyl sulfoxide (DMSO) and *N*,*N*-dimethyl formamide (DMF) and other reactants were purchased from commercial suppliers and used without further purification.

### 3.2. Characterization Techniques

Thermogravimetric measurements (TGA) were carried out on a 10 to 20 mg sample with a TGA Q500 apparatus (TA Instruments, New Castle, DE, USA) at a scan rate of 5 °C⋅min^−1^ from 40 to 800 °C under dynamic air atmosphere at a flow rate of 40 mL⋅min^−1^.

Differential scanning calorimetry (DSC) experiments were carried out on a TA Instruments DSC Q20. The measurements were performed by heating around 10 mg of samples from −70 °C to 180 °C for hybrid material and commercial PEO. The heating rate was 10 °C min^−1^. The melting temperature (T_m_) and the enthalpies of melting ∆H_m_ were all extracted from the second heating scan. The degree of crystallinity ($X_{c}$) was calculated according to the following equation:

(1) $$X_{c}=\frac{∆H_{m}}{w_{PEO}*∆H_{m}^{o}}*100\%$$

where $w_{PEO}$ is the weight fraction of PEO in the LPSQ hybrid materials, ΔH_m_ is the measured enthalpy of melting of pristine or grafted PEO and $∆H_{m}^{o}$ is the enthalpy of fusion of a 100% crystalline PEO taken as 195 J g^−1^ [34].

Wide-angle X-ray scattering (WAXS) experiments were performed on SAXSess-MC2 (Anton-Paar, GmbH, Vienna, Austria) with a sealed copper tube as the X-ray source (wavelength is 0.15417 nm (Cu K-α)) equipped with a Kratky block collimation system and using an image plate (IP 200 × 60 mm, pixel resolution 50 × 50 μm) as the detector. The system operates in line collimation, which allows achieving higher scattering intensities and images. Plates were read with the Cyclone (Perkin Elmer, Shelton, CT, USA) and Optiquant 3.0 software system. An exposure time of 30 min was long enough to provide a good signal-to-noise ratio (scattering angle range 0.1 < q < 28 nm^−1^). SAXSquant 3.5 was used as data analysis software.

Fourier-transform infrared (FTIR) analysis was performed on a Perkin Elmer Two FTIR Spectrometer with an ATR accessory.

All solid-state Cross Polarization Magic Angle Spinning (CPMAS) NMR spectra were obtained on a Bruker (Billerica, MA, USA) Avance-400 MHz NMR spectrometer operating at a ^13^C and ^29^Si resonance frequency of 101.6 MHz and 79.5 MHz, respectively. ^13^C and ^29^Si CPMAS experiments were performed with a commercial Bruker double-bearing probe. About 100 mg of samples was placed in zirconium dioxide rotors of 4 mm outer diameter and spun at a Magic Angle Spinning rate of 10 kHz. The CP technique [35] was applied with a ramped 1H pulse starting at 100% power and decreasing until 50% during the contact time in order to circumvent Hartmann–Hahn mismatches [36,37]. The contact times were 2 ms for ^13^C CPMAS and 5 ms for ^29^Si CPMAS. To improve the resolution, a dipolar decoupling GT8 pulse sequence was applied during the acquisition time [38]. To obtain a good signal-to-noise ratio, 6144 scans were accumulated using a delay of 2 s in the ^13^C CPMAS experiment, and 4096 scans with a delay of 5 s in the ^29^Si CPMAS experiment. The ^13^C and ^29^Si chemical shifts were referenced to tetramethylsilane.

Viscoelastic properties of the LPSQ hybrid or PEO and PMMA composite were studied using an Anton Paar Rheometer MCR 302 equipped with 8 mm diameter aluminum parallel disks. The polymer was pressed to a gap width of 0.9 mm. The complex shear modulus, G* = G’ + iG”, was then measured as a function of frequency by dynamically shearing the compound at a fixed strain of 1% over the frequency range 0.1–100 rad s^−1^ at 80 °C for the PEO composite and 150 °C for the PMMA composite. All measurements were performed in a dry air atmosphere.

Size exclusion chromatography (SEC) analysis was performed on an apparatus from Agilent Technologies (Santa Clara, CA, USA), which was equipped with a 1260 infinity pump, a 1260 infinity autosampler, a 1260 infinity UV photodiode array detector and a 1260 infinity RI detector. The stationary phase was composed of two PSS-SDV Linear M columns and a precolumn thermostated at 40 °C. Mobile phase was THF at 1 mL.min^−1^. Samples were prepared at a concentration of 0.25 wt.% in THF containing 0.25 vol.% of toluene, as a flow marker. Injection volume was 20 μL. Polystyrene equivalent number-average and weight-average molar masses (M_n_ and M_w_) and dispersities *Ɖ* were calculated by means of PS calibration curve using PS-M Easivial (Agilent, USA).

### 3.3. Synthesis of Chloropropyl Ladder PolySilsesQuioxane (***2a***)

A solution of K_2_CO_3_ (20 mg, 0.144 mmol, 0.0036 eq) in water (2.6 mL) was added to a solution of (3-chloropropyl) triethoxysilane (9.6 g, 40 mmol, 1 eq) in THF (5.2 mL). Then, MeOH (800 µL) was added. The mixture was stirred at room temperature for 9 days. After removing THF under vacuum, the mixture was diluted with CH_2_Cl_2_ (40 mL) and water (10 mL). The organic phase was isolated and dried over MgSO_4_; the solution was evaporated to give the final product **2a** (5 g, yield > 95%). ^1^H NMR (300 MHz, CDCl_3_) δ: 3.55 (broad peak, 2H, CH_2_-Cl), 1.85 (broad peak, 2H, CH_2_), 0.80 (broad peak, 2H, CH_2_-Si).^13^C NMR (75 MHz, CDCl_3_) δ: 47.1 (CH_2_-Cl), 26.5 (CH_2_), 10.6 (broad, Si-CH_2_). An amount of 4 mol% of ethoxy signal was still present, because of remaining unhydrolyzed functions. ^29^Si CPMAS NMR: −58.3 (T^2^), −68.5 (T^3^). IR asymmetric stretching vibrations of the perpendicular bonds between the silicon and the oxygen of the LPSQ were at 1025.5 cm^−1^ and 1102.5 cm^−1^. SEC analysis gave an M_n_ of 4 200 g mol^−1^ and a dispersity (Ð) of 2.48.

### 3.4. Synthesis of Chloropropyl Ladder PolySilsesQuioxane (***2b***)

A solution of K_2_CO_3_ (10 mg, 0.0725 mmol, 0.0036 eq) in water (1.3 mL) was added to a solution of (3-chloropropyl) trimethoxysilane (4 g, 20.129 mmol, 1eq) in THF (2.6 mL). The mixture was stirred at room temperature for 18 h. After removing THF under vacuum, the mixture was diluted with CH_2_Cl_2_ (30 mL) and water (10 mL). The aqueous phase was eliminated, and the organic phase was dried over MgSO_4_ and filtered; the solution was evaporated to give the final product **2b** (668 mg, 26%). ^1^H NMR (300 MHz, CDCl_3_) δ: 3.54 (broad peak, 2H, CH_2_-Cl), 1.85 (broad peak, 2H, CH_2_), 0.80 (broad peak, 2H, CH_2_-Si).^13^C NMR (75 MHz, CDCl_3_) δ: 47.0 (CH_2_-Cl), 26.4 (CH_2_), 9.9 (broad, Si-CH_2_). Infrared asymmetric stretching vibrations of the perpendicular bonds between the silicon and the oxygen of the LPSQ were at 1028 cm^−1^ and 1099 cm^−1^. SEC analysis gave a number-average molecular weight (M_n_) of 3000 g mol^−1^ and a dispersity (Ð) of 2.7 based on polystyrene standards calibration. ^29^Si CPMAS NMR: −58.3 (T^2^), −68.5 (T^3^).

### 3.5. Synthesis of Azidopropyl Ladder PolySilsesQuioxane (N_3_-LPSQ or ***3***)

NaN_3_ (527 mg, 8.101 mmol, 1.5 eq) was added to a solution of **2a** (700 mg, 5.4 mmol, 1 eq) in DMF (7 mL). The reaction was stirred at 40 °C under an Argon atmosphere for 3 days. The mixture was diluted with CH_2_Cl_2_ (150 mL) and washed with water (400 mL). The aqueous phase was isolated and extracted with CH_2_Cl_2_ (20 mL × 5). The organic phases were combined and dried over MgSO_4_; the solution was then evaporated to give the **N_3_-LPSQ** (**3**) (631 mg, 4.64 mmol, 86%).

^1^H NMR (300 MHz, CDCl_3_) δ: 3.28 (broad peak, 2H, CH_2_-N_3_), 1.68 (broad peak, 2H, CH_2_), 0.74–0.70 (broad peak, 2H, CH_2_-Si). ^13^C NMR (75 MHz, CDCl_3_) δ: 53.5 (CH_2_-N_3_), 22.7 (CH_2_), 9.6 (Si-CH_2_). ^29^Si CPMAS NMR: −57.5 (T^2^), −67.7 (T^3^). IR asymmetric stretching vibrations of the perpendicular bonds between the silicon and the oxygen of the LPSQ were the two bands at 1039 cm^−1^ and 1096 cm^−1^ and N_3_ elongation band at 2090 cm^−1^.

### 3.6. Synthesis of Propargyl-Functionalized Poly (Ethylene Glycol) (PEG)

- *Synthesis of propargyl-PEG of 1000 g mol^−1^* (**4**)

In a 100 mL flask, polyethylene glycol monomethyl ether of 1000 g mol^−1^ (PEG1) (4.9 g, 4.9 mmol, 1 eq) was dissolved in anhydrous THF (4.9 mL) and cooled down to 0 °C. Under argon, the NaH 60% dispersed in oil (392 mg, 9.8 mmol, 2 eq) was introduced to the previous solution. The mixture was stirred at 0 °C for 1 h before adding in dropwise a solution of propargyl bromide 80% in toluene (2.73 mL, 24.5 mmol, 5 eq). After 24 h stirring at 30 °C, DCM was added, and the mixture was filtered. The filtrate was concentrated under vacuum; the resulting solid was crushed in cold ethyl ether, filtered and dried under vacuum. Propargyl-PEG1 (**4**) was obtained quantitatively as 5 g of a beige solid.

^1^H NMR (300 MHz, CDCl_3_) δ: 4.20 (m, 2H, propargyl CH_2_), 3.67 (broad peak, 82H, OCH_2_), 3.56 (m, 2H, OCH_2_ next to OCH_3_ end group), 3.39 (s, 3H, OCH_3_), 2.45 (m, 1H, CH).

- *Synthesis of propargyl-PEG of 5000 g mol^−1^* (**5**)

In a 100 mL flask, dried polyethylene glycol monomethyl ether of 5000 g mol^−1^ (PEG5) (10 g, 2 mmol, 1 eq) was dissolved in anhydrous THF (10 mL) at 60 °C and cooled down to 40 °C. Under argon, the NaH 60% dispersed in oil (120 mg, 3 mmol, 1.5 eq) was introduced slowly to the previous solution. The mixture was stirred at 40 °C for 1 h before adding in dropwise a solution of propargyl bromide 80% in toluene (664 μL, 6 mmol, 1.5 eq). After 48 h stirring at 40 °C, the warm mixture was filtered and rinsed with DCM. The filtrate was concentrated under vacuum and the concentrated solution was precipitated in cold diethyl ether. The solid was filtered and dried under vacuum. Propargyl-PEG5 (**5**) was obtained quantitatively as a beige solid.

^1^H NMR (300 MHz, CDCl_3_) δ: 4.19 (m, 2H, propargyl CH_2_), 3.63 (m and broad peak, 452H, OCH_2_), 3.36 (s, 3H, OCH_3_), 2.43 (m, 1H, CH).

### 3.7. Grafting of PEG to the LPSQ by “Click Chemistry”

Two methods were used to graft PEG to the PLSQ. For the short PEG of 1000 g mol^−1^, the click reaction was catalyzed by copper(I) while for the longer one (PEG of 5000 g mol^−1^), the click reaction was carried out without catalyst at the melting state.

- *Click reaction catalyzed by copper (I)*—**PEG1-LPSQ** (**6**)

A solution of propargyl-PEG1 (**4**) (2.29 g, 2.205 mmol, 1 eq) in THF/H_2_O (11.3 mL/5.6 mL) and LPSQ (300 mg, 2.205 mmol, 1 eq) was stirred at RT for 10 min. Then, CuSO_4_.5H_2_O (275 mg, 1.102 mmol, 0.5 eq) and an aqueous solution of sodium ascorbate (218 mg, 1.102 mmol, 0.5 eq. in 3 mL H_2_O) were added. The resulting mixture was stirred at RT. The reaction was monitored by ^1^H NMR in CDCl_3_ following the disappearance of the propargylic proton at 2.4 ppm.

After 3 days, because of the remaining residual propargyl signal, a second addition of a solution of sodium ascorbate (218 mg, 1.102 mmol, 0.5 eq in a minimal amount of water) and CuSO_4_.5H_2_O (275 mg, 1.102 mmol, 0.5 eq) was performed. Two other portions of catalyst were required and added to the reactional mixture on the 6th and 9th day to reach the complete consummation of propargyl-PEG1.

After 11 days, THF was evaporated and the 2.5 g crude solid (green flakes) was dissolved in 15 mL of a mixture of acetone/water (2:1, *v/v*) under ultrasounds. This solution was then dialyzed against EDTA solution (400 mg L^−1^) and deionized water to eliminate residual cupper ions and non-reacted LPSQ using tubing membrane with a cut off of 6000–8000 Da. After evaporation of water and drying in the vacuum, a beige solid was obtained called **PEG1-LPSQ** (**6**) (700 mg, 1.00 mmol, 45 wt%) as the single *Z* isomer. ^13^C CPMAS NMR: (170 (spinning side bands)), 143.5 (Cq triazole), 123.1 (CH triazole), 70.9 (OCH_2_, broad, PEG), 69.5 (CH_2_), 57.5 (OCH_3_), 50.9 (CH_2_N), 22.9 (CH_2_), 8.5 (CH_2_-Si). ^29^Si CPMAS NMR: −57.7 (T^2^), −66.9 (T^3^). FTIR: 2868 cm^−1^ (OCH_2_ of PEG chain); N_3_ function seems to be masked as only a very small peak was observed at 2089 cm^−1^ (Figure S6a, Supplementary Materials).

- *Click reaction in thermal condition without catalyst*—**PEG5-LPSQ** (**7**)

Propargyl-PEG5 (**5**) (741 mg, 0.147 mmol, 0.1 eq) was dried under vacuum for 3 h at 100 °C in order to eliminate water (PEG5 melts at 60 °C). Under argon atmosphere and under stirring, the **N_3_-LPSQ** (**3**) (200 mg, 1.470 mmol, 1 eq) was added to the melt PEG. The reaction was monitored by ^1^H NMR in CDCl_3_ by following the disappearance of the propargylic proton signal of **5** located at 2.4 ppm. The reaction was carried out under stirring at 130 °C for 2 days. After cooling to RT, the solid crude product (940 mg) was dissolved in 15 mL of a mixture of acetone/water (2:1, *v/v*) under ultrasounds. This solution was then dialyzed against deionized water to eliminate non-reacted compounds using tubing membrane with a cut off of 25,000 Da. After evaporation of water and drying in the vacuum, a beige solid was obtained called **PEG5-LPSQ** (**7**) (800 mg, 85%wt). The NMR analysis indicates the formation of the 2 position isomers from cycloaddition 1–3 dipolar in 50/50 ratio. ^13^C CPMAS NMR: (170 (spinning side bands) and 145.4 (Cq of the 2 triazole isomers), 124.7 and 134.5 (CH of the 2 triazole isomers), 71.0 (OCH_2_, broad, PEG), 64.9 (CH_2_), 61.5 (OCH_3_), 54.2 (CH_2_N), 23.5 (CH_2_), 10.7 (CH_2_-Si). ^29^Si CPMAS NMR: −57.5 (T^2^), −67.4 (T^3^). FTIR: 2876.2 cm^−1^ (OCH_2_ of PEG chain); N_3_ elongation band at 2098 cm^−1^, 1097 cm^−1^ and 1060 cm^−1^ for siloxane Si-O-Si.

### 3.8. Preparation of Composite Materials from Hybrid Materials (PEG1-LPSQ (***6***) and PEG5-LPSQ (***7***)) Dispersed in 100K PEO and 89K PMMA Matrices

Composites were prepared by the solvent casting process. They contained PEG-grafted LPSQ as the nanofiller and either PEO or PMMA as the matrices. The weight fraction of silica (SiO_1.5_) in the composite was fixed at 2.5 wt% or 1 wt%. In a general protocol, the solutions of hybrid materials (**PEG1-LPSQ** (**6**) or **PEG5-LPSQ** (**7**) and polymer matrices (PEO or PMMA) were prepared separately in DCM or DMSO. The resulting solutions were then mixed together and stirred for 24 h. The solvent was evaporated at 60 °C under vacuum until a constant weight. Several samples were prepared, and the experimental data are summarized in Table 2. The resulting composites were labeled “*M-Qx-y*”, where *M* corresponds to the polymer matrix (PEO or PMMA), *Qx* corresponds to the LPSQ functionalized with PEG, *x* = 1 or 5 corresponding to the grafted PEG of 1000 g mol^−1^ or 5000 g mol^−1^, respectively, and *y* corresponds to the weight fraction of silica in the composite materials.

## 4. Conclusions

In the present work, we have successfully synthesized two LPSQ molecules from either 3-chloropropyl triethoxysilane or 3-chloropropyl trimethoxysilane. According to the characterization results obtained by NMR, FTIR, WAXS and SEC, the LPSQ (**2a**) prepared from 3-chloropropyl triethoxysilane showed a better-defined structure than the other one because of the fast hydrolysis of the trimethoxysilane compound. The functionalization of LPSQ with PEG chains (M_n_ of 1000 g mol^−1^ and 5000 g mol^−1^) was performed via click chemistry between azide groups of the functionalized LPSQ structure and propargyl ether on the PEG chains. Two click conditions were experimented, and both led to the expected PEG-grafted LPSQ hybrid materials. The latter were used as nanofiller to elaborate nanocomposite materials using either PEO or PMMA as matrix. In the case of PEO-based nanocomposites, for the same silica (SiO_1.5_) content (e.g., 2.5 wt%), DSC analysis revealed that whatever the M_n_ of grafted PEG chains, T_m_ and crystallinity degree of the resulting nanocomposites shifted just a little, while rheology analysis showed a strong effect of M_n_ of grafted PEG chains on the viscoelastic properties of their nanocomposites. Indeed, PEG(1 kDa)-grafted LPSQ conferred a reinforcement character to the composite while PEG(5 kDa)-grafted LPSQ acted as a plasticizer, attested by the lower G’ and G” modulus values of PEO-Q5-2.5 composite compared to those of PEO matrix. Consequently, if we are looking for the reinforcement effect of PEO matrix, it would be more relevant to use short-PEG-chain-grafted LPSQ than long-PEG-chain-grafted LPSQ. Concerning PMMA-based nanocomposites, two T_g_ values were observed for PMMA-Q1-2.5 composite with 32 wt% of PEG, indicating a partial miscible blend, while only one T_g_ value was obtained for PMMA-Q5-2.5 composite with 10 wt% of PEG, which is proof of a miscible blend. This observation is consistent with the literature results where the low limit of PEO content for a miscible PEO/PMMA blend was around 25 wt%. The fact that the PEG chains are grafted does not change the miscibility behavior of PEG-grafted LPSQ/PMMA blends. However, the thermal properties such as T_g_ and T_m_ of PMMA-Q5-2.5 composite were strongly affected, as well as its viscoelastic properties. Indeed, the obtained T_g_ value (T_g_ = −13 °C) was far from the expected value (T_g_ around 90 °C) given in the literature [15,28], while G’ and G” modulus values were one and a half decade lower than that of the pure PMMA matrix. The decrease in viscoelastic modulus of the PMMA-based composite is owing to the presence of PEG-grafted chains, which act as a plasticizer for PMMA. Albeit less pronounced, this effect was also observed for the partially miscible blend PMMA-Q1-2.5. The study of the thermodynamic and viscoelastic behavior of composites containing polymer-grafted LPSQ should constitute a fascinating field of investigation, while the PEO- or PMMA-based composites containing PEG-grafted LPSQ should lead to interesting solid electrolytes for lithium battery applications.

## Figures and Tables

**Table 1:** Thermal characteristics of PEG/PEO, PEG-grafted LPSQ, PEO-based and PMMA-based composites.

Entry	Sample	T_g_ (°C)	T_m_ (°C)	ω_PEG (PEO)_ ^a^	ΔH_m_ (J g^−1^)	X_c_ (%)
1	PEG1 ^b^		38.7	1.00	166.8	85.5
2	PEG5 ^b^		64.3	1.00	172.1	88.3
3	PEO ^c^		65.6	1.00	146.7	75.2
4	PEG1-LPSQ (**6**)		36.5	0.80	66.3	42.5
5	PEG5-LPSQ (**7**)		57.3	0.61	83.2	69.9
6	PEO-Q1-2.5	−45.9	25.4 54.6	0.32 (0.60)	9.6 69.9	15.4 59.7
7	PEO-Q5-2.5	−51.2	57.9	0.10 (0.83)	142.1	78.4
8	PEO-Q5-1.0	−58.7	52.5	0.04 (0.93)	130.4	68.9
9	PMMA-Q1-2.5	−43.1 97.4		0.32	23.6	37.7
10	PMMA-Q5-2.5	−13.2		0.10	1.2	6.0

^a^ weight fraction of PEG and PEO in the materials. ^b^ PEG1 and PEG5 are pristine PEG of molar masses of 1000 g mol^−1^ and 5000 g mol^−1^. ^c^ PEO of molar mass of 100,000 g mol^−1^ used as matrix for the preparation of composites.

**Table 2:** Experimental data for the preparation of composite materials.

Composite	Nanofiller (mg)	m_nanofiller_ (mg)	Polymer Matrix	m_matrix_ (mg)	wt% SiO_1.5_ in Composite
PEO-Q1-2.5	PEG1-LPSQ (**6**)	51.6	PEO	78.4	2.5
PMMA-Q1-2.5	PEG1-LPSQ (**6**)	51.6	PMMA	78.4	2.5
PEO-Q5-1	PEG5-LPSQ (**7**)	10.1	PEO	139.1	1.0
PEO-Q5-2.5	PEG5-LPSQ (**7**)	25.3	PEO	124.7	2.5
PMMA-Q5-2.5	PEG5-LPSQ (**7**)	25.3	PMMA	124.7	2.5

## Data Availability

The data that support the findings of this study are available from the corresponding author upon reasonable request.

## Supplementary Materials

The following supporting information can be downloaded at: https://www.mdpi.com/article/10.3390/molecules29245832/s1, Figure S1: ^29^Si spectrum of LPSQ **2a**; Figure S2: ^29^Si spectrum of LPSQ **2b**; Figure S3: infrared spectrum of LPSQ **2a**; Figure S4: TGA thermogram of PEG1-LPSQ (**6**) and associated calculation of the click reaction yield; Figure S5: TGA thermogram of PEG5-LPSQ (**7**) and associated calculation of the click reaction yield; Figure S6: FTIR spectra of (a) PEG1-LPSQ (**6**) and (b) PEG5-LPSQ (**7**); Figure S7: ^13^C NMR spectra of (a) PEG1-LPSQ (**6**) and (b) PEG5-LPSQ (**7**); Figure S8: DSC thermograms during the second heating run of pristine PEG1 and PEG1-grafted LPSQ (**6**); Figure S9: DSC thermograms during the second heating run of pristine PEG5 and PEG5-grafted LPSQ (**7**); Figure S10: DSC thermogram during the second heating run of PMMA matrix of 89,000 g mol^−1^; Figure S11: DSC thermogram during the second heating run of PMMA-Q1-2.5 composite; Figure S12: DSC thermogram during the second heating run of PMMA-Q5-2.5 composite; Figure S13: Tan δ versus frequency of pure PEO 100 kDa and its nanocomposites.
