# Supplementary material for: Organic–Inorganic Hybrid Ladder-like Polysilsesquioxanes as Compatibilized Nanofiller for Nanocomposite Materials
Source: Molecules. 2024 Dec 11;29(24):5832. doi: 10.3390/molecules29245832 (PMC11728607; doi:10.3390/molecules29245832)
Supplement: Supplementary file 1 [file molecules-29-05832-s001.zip › molecules-3318926-supplementary.pdf]

Supplementary Materials

Organic–Inorganic Hybrid Ladder-like Polysilsesquioxanes as Compatibilized Nanofiller for Nanocomposite Materials

Dominique Mouysset [1], Marion Rollet[1], Emily Bloch [2], Stéphane Gastaldi [1], Eric Besson [1,*], Trang N. T. Phan [1,*]

[1] Aix Marseille Univ, CNRS, Chemistry Department, Institute of Radical Chemistry (ICR), 13397 Marseille, France.
[2] Aix Marseille Univ, CNRS, Chemistry Department, Laboratory of Divided Materials, Interfaces, Reactivity, Electrochemistry (MADIREL), 13397 Marseille, France.

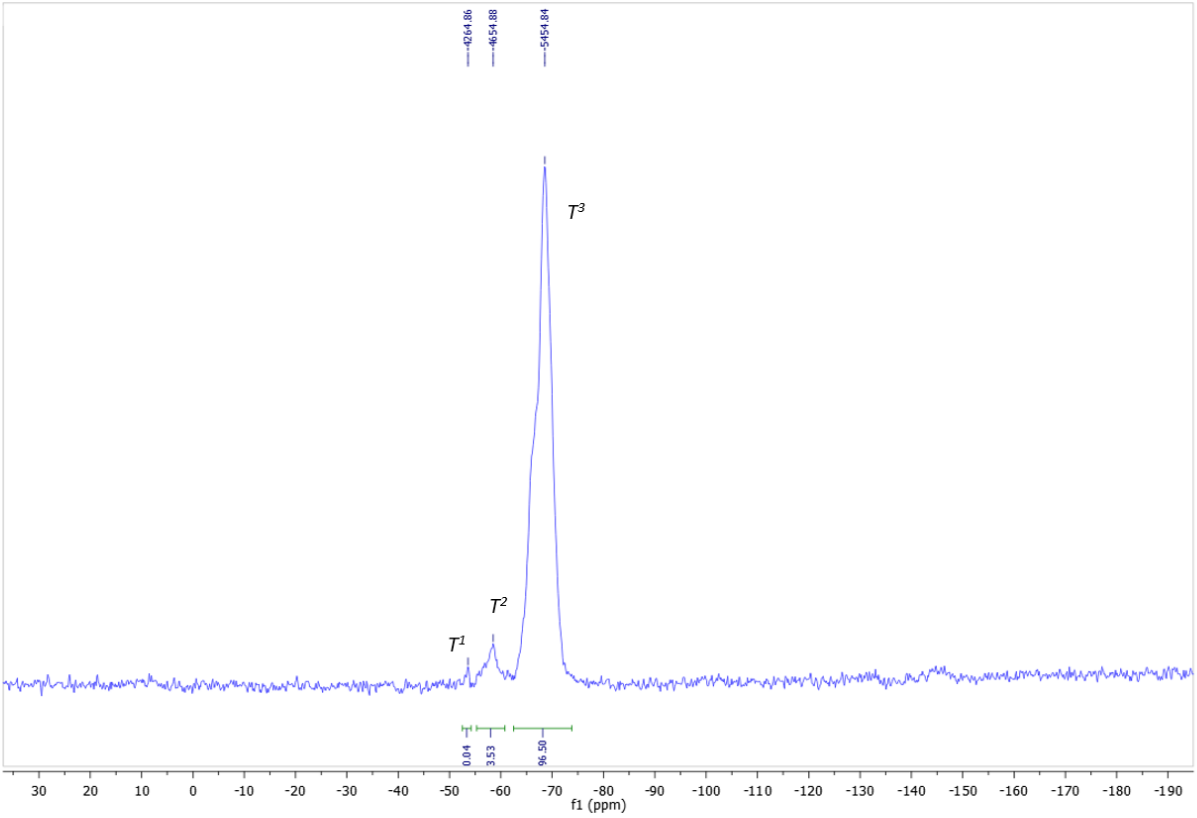

Figure S1 : ^{29}Si spectrum of LPSQ **2a**

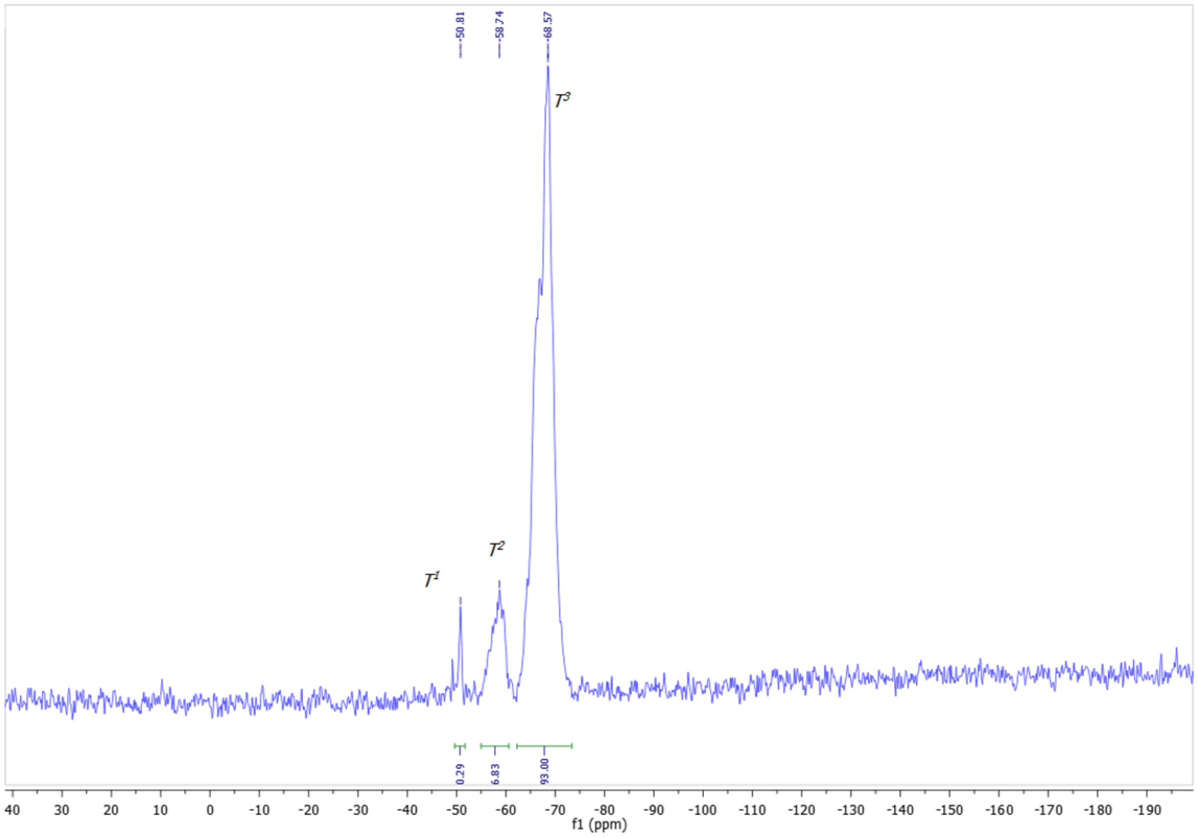

Figure S2 : ^{29}Si spectrum of LPSQ **2b**

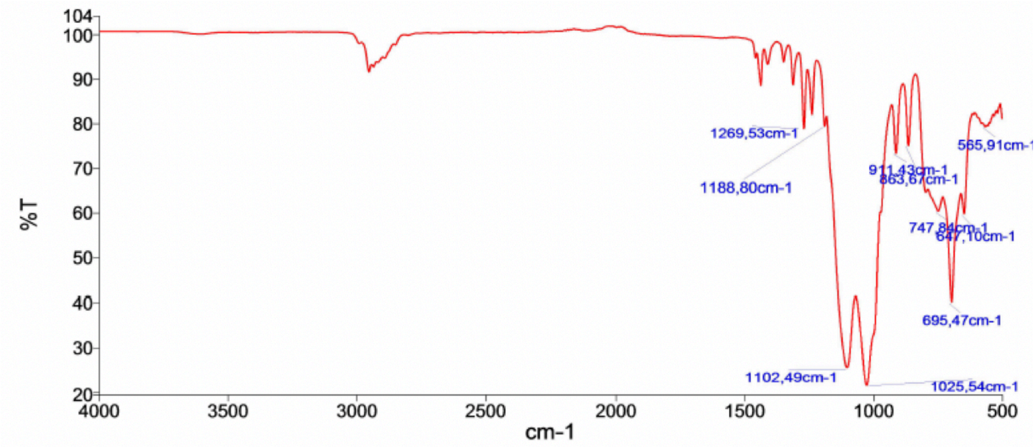

Figure S3: IR spectrum of LPSQ **2a**

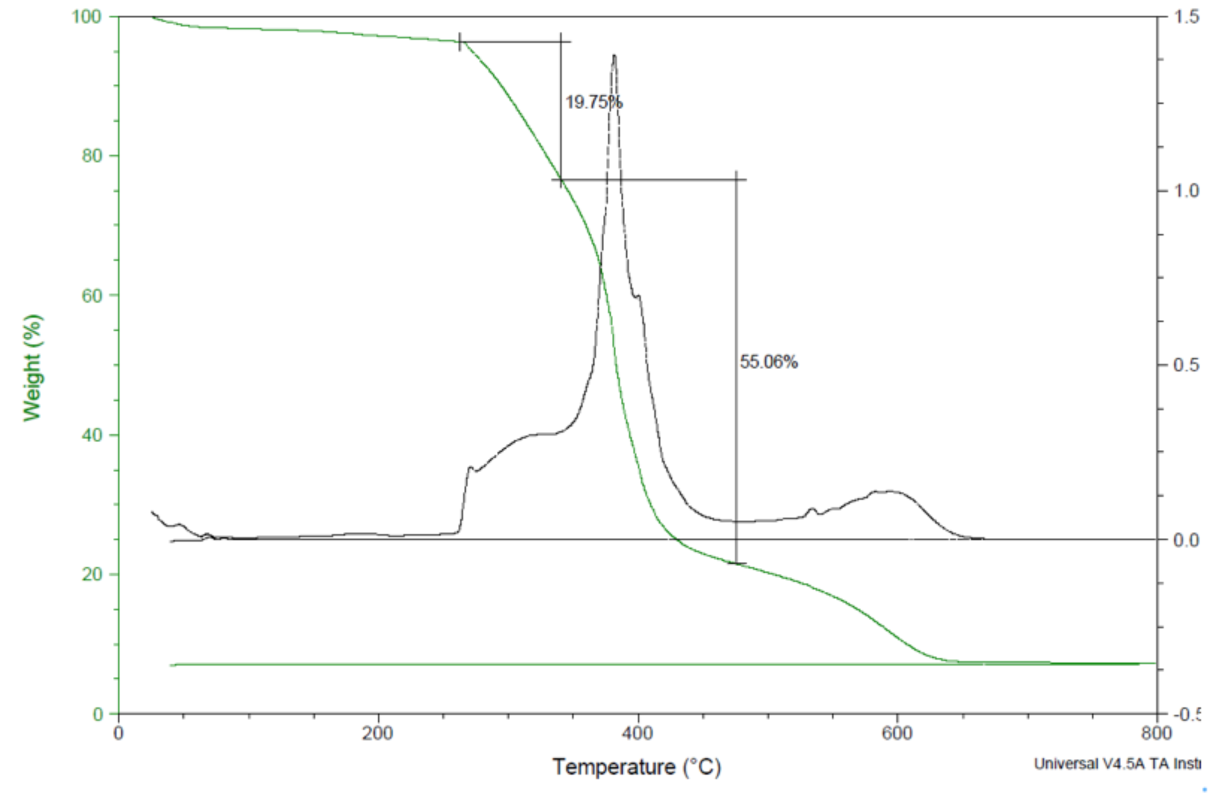

Figure S4 : TGA thermogram of PEG1-LPSQ (**6**)

Calculation of the click reaction yield (x) in the grafting of propargyl-PEG1 (4) to azide-LPSQ (3)

The weight loss (WL) given by TGA between 180°C and 700°C is 92.5%

WL = [MW propylPEG(x) + MWpropylN_3 (1-x)] / [MW$SiO_{1.5}$propyl-PEG (x) + MW$SiO_{1.5}$ propylN_3 (1-x)]

where x is the molar fraction of propyl-PEG

WL = 0.925 = [1084(x) + (1-x)84] / [1136(x) +(1-x)136]

x = 0.56

so the yield of click reaction was 56 mol%

Calculation of the weight fraction of PEG (ω) in the hybrid PEG1-LPSQ (6)

0.56 x $MW_{LPSQPEO}$ = 0.56 x 1136 = 636 g/mol in hybrid material

0.44 x MW_{LPSQN3} = 0.44 x 136 = 59.8 g/ mol in hybrid material

MW(PEG1-LPSQ) = 695.8 g/mol

ω (PEG) in PEG1-LPSQ = (0.56 x 1000) / 695.8 = 0.8

ω ($SiO_{1.5}$) in PEG1-LPSQ = (1 x 52) / 695.8 = 0.07

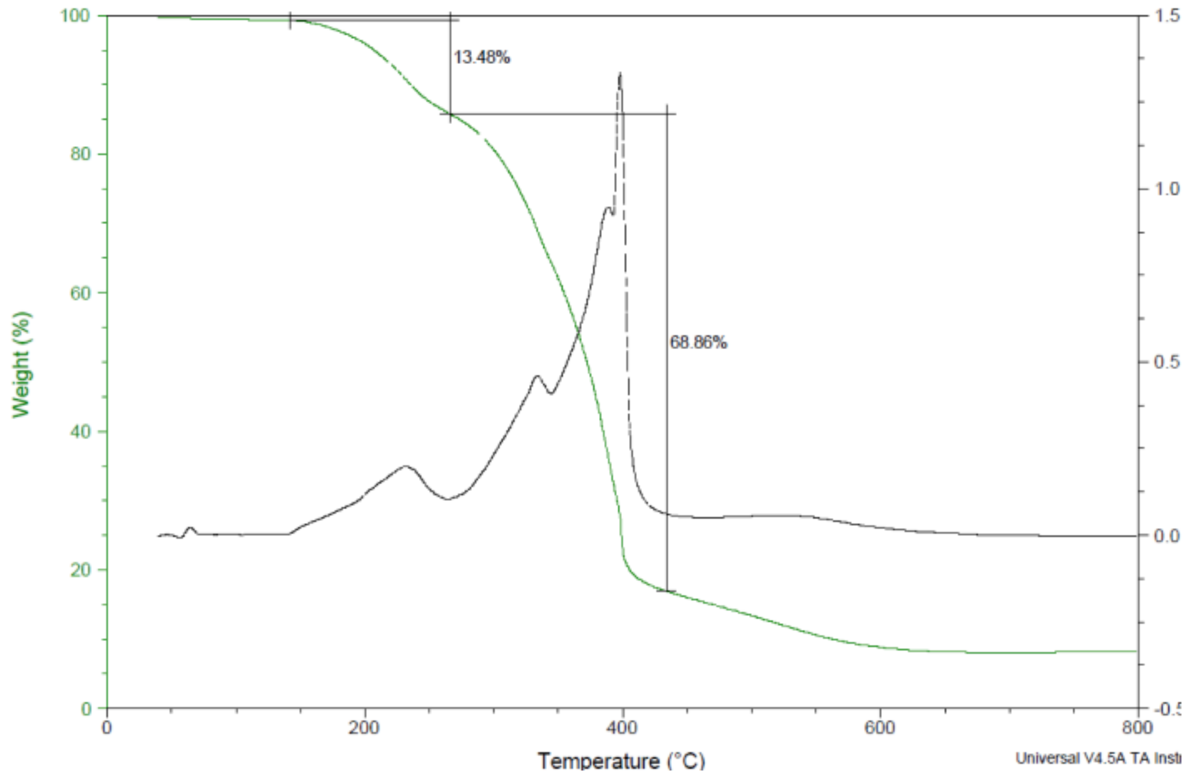

Figure S5 : TGA thermogram of PEG5-LPSQ (7)

Calculation of the click reaction yield (x) in the grafting of propargyl-PEG5 (5) to azide-LPSQ (3)

The weight loss (WL) given by TGA between 180°C and 700°C is 91.84%

WL = [MW propylPEG(x) + MWpropylN_3 (1-x)] / [MWSi$O_{1.5}$propyl-PEG (x) + MWSi$O_{1.5}$ propylN_3 (1-x)]

where x is the molar fraction of propyl-PEG

WL = 0.918 = [5084(x) + (1-x)84] / [5136(x) +(1-x)136]

x = 0.043

so the yield of click reaction was 4.3 mol%

Calculation of the weight fraction of PEG (ω) in the hybrid PEG5-LPSQ (7)

0.043 x $MW_{LPSQPEO}$ = 0.043 x 5136 = 220.8 g/mol in hybrid material

0.957 x MW_{LPSQN3} = 0.957 x 136 = 130 g/ mol in hybrid material

MW(PEG5-LPSQ) = 350.95 g/mol

ω (PEG) in PEG5-LPSQ = (0.043 x 5000) / 350.95 = 0.610

ω (Si$O_{1.5}$) in PEG5-LPSQ = (1 x 52) / 350.95 = 0.148

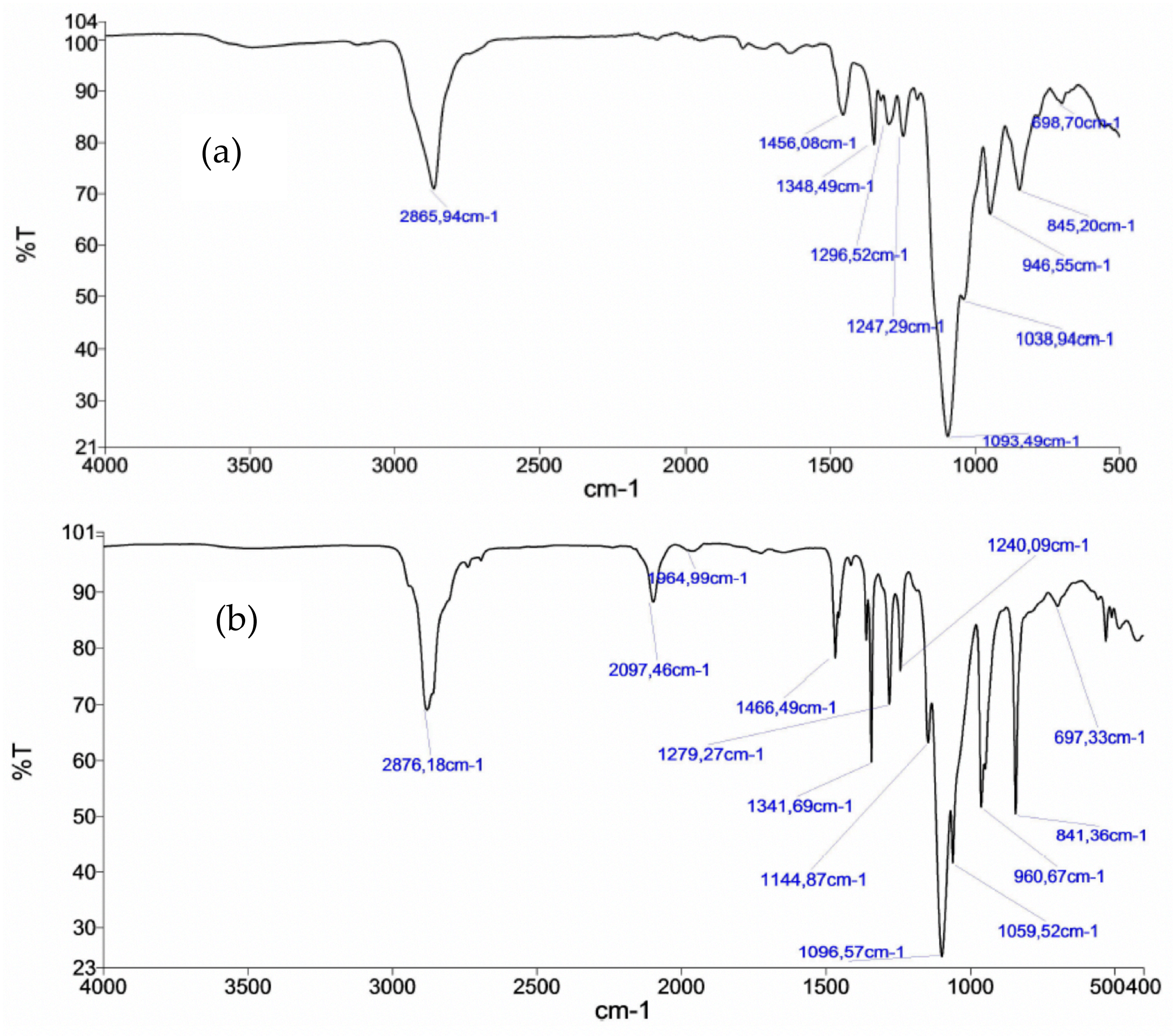

Figure S6 : FTIR spectra of (a) PEG1-LPSQ (**6**) and (b) PEG5-LPSQ (**7**)

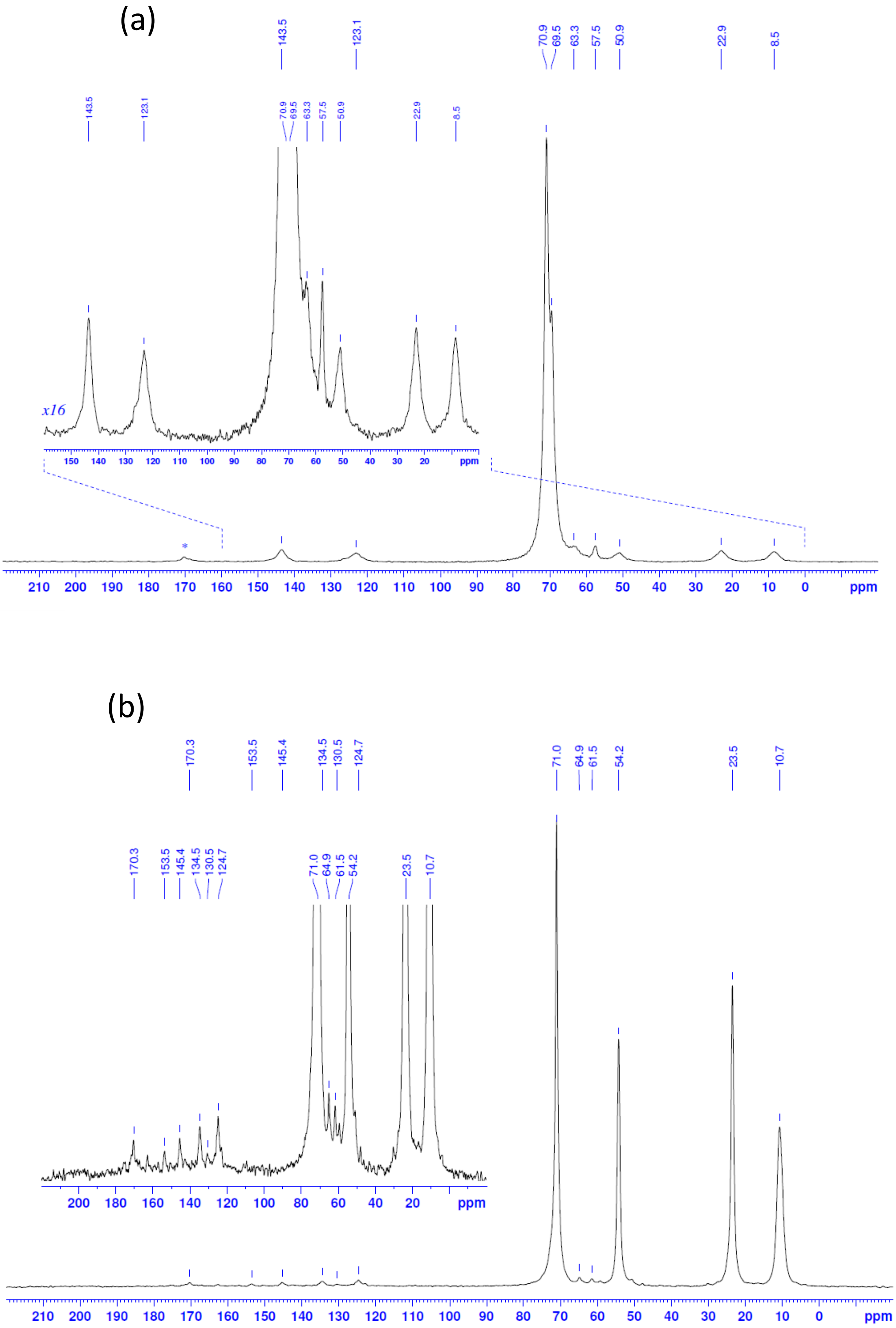

Figure S7 : ^{13}C NMR spectra of (a) PEG1-LPSQ (**6**) and (b) PEG5-LPSQ (**7**)

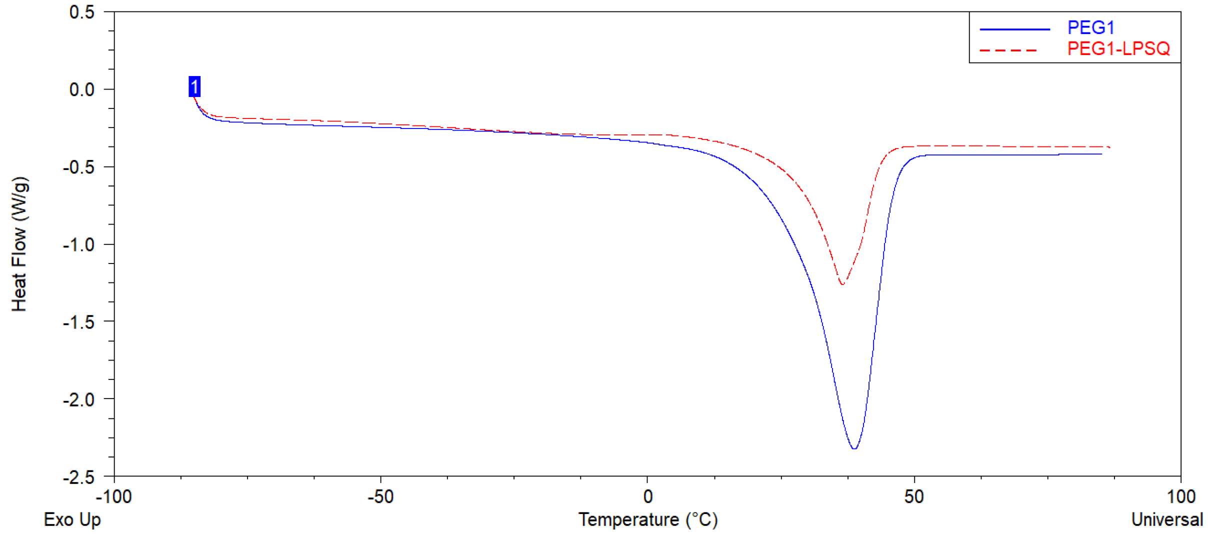

Figure S8 : DSC thermograms during the second heating run of pristine PEG1 and PEG1-grafted LPSQ (**6**)

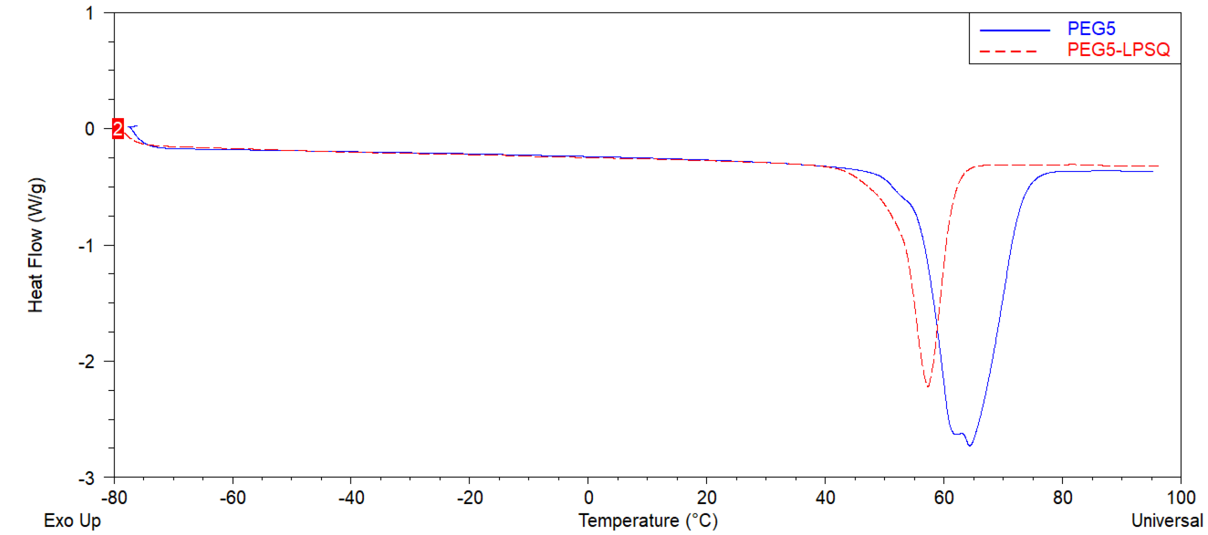

Figure S9 : DSC thermograms during the second heating run of pristine PEG5 and PEG5-grafted LPSQ (**7**)

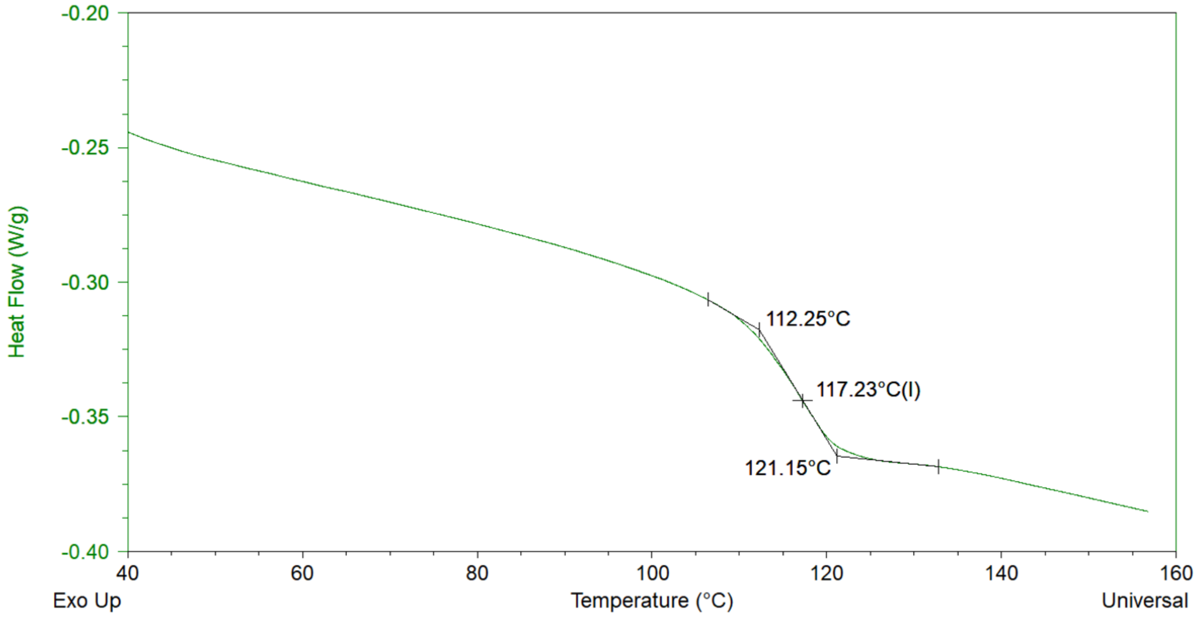

Figure S10 : DSC thermogram during the second heating run of PMMA matrix of 89 000 g.mol^{-1}.

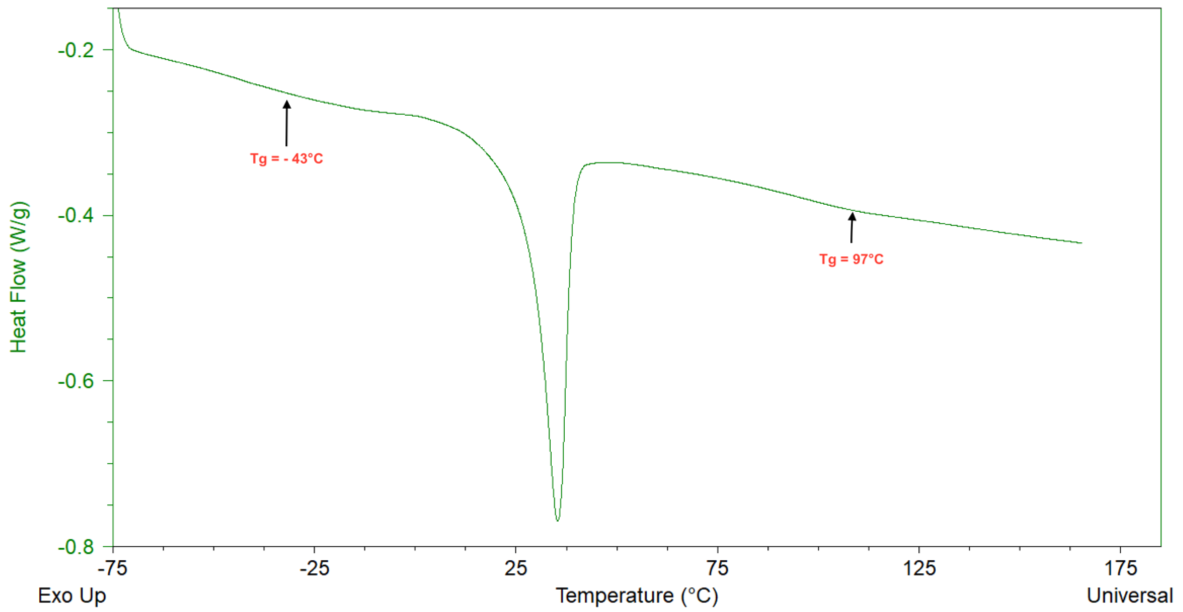

Figure S11 : DSC thermogram during the second heating run of PMMA-Q1-2.5 nanocomposite.

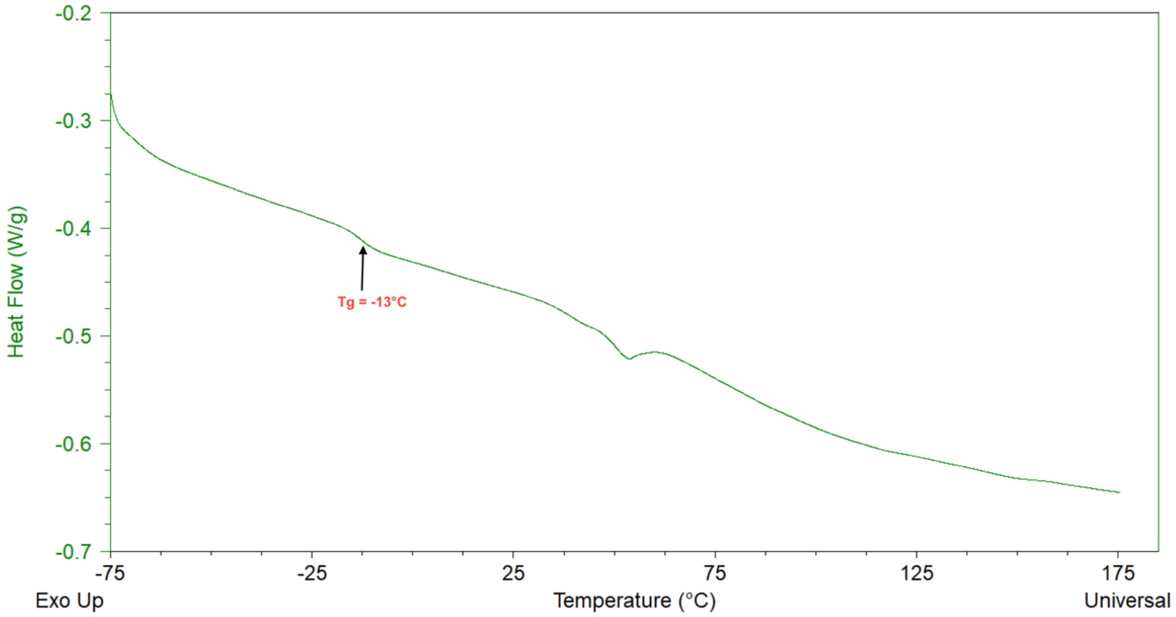

Figure S12 : DSC thermogram during the second heating run of PMMA-Q5-2.5 nanocomposite.

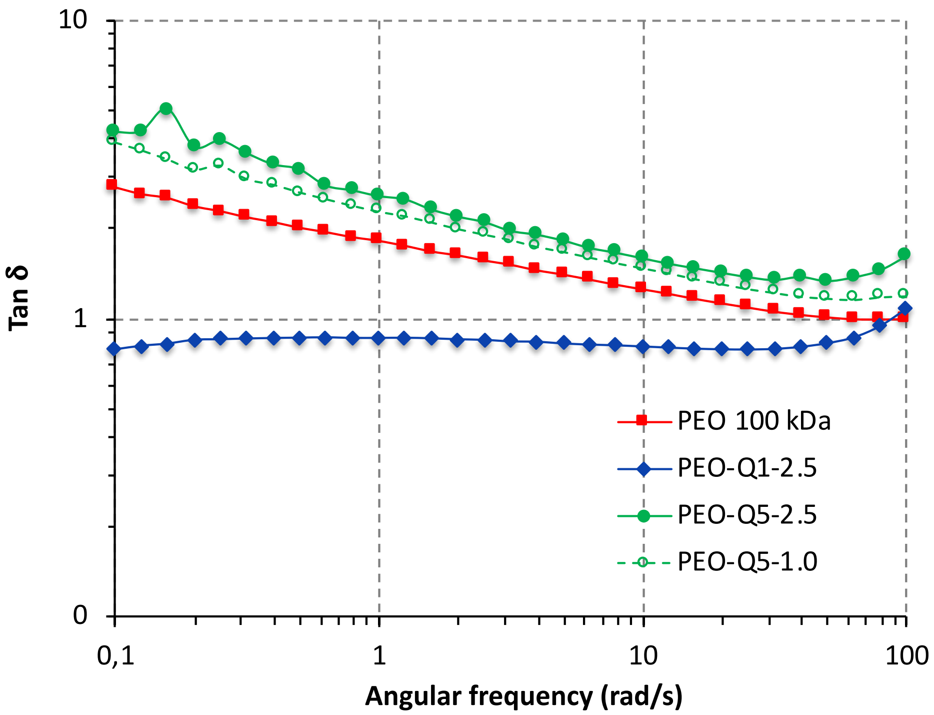

Figure S13 : Tan δ *versus* frequency of pure PEO 100 kDa and its nanocomposites.
